# Traditional Chinese medicine strategies to optimize antibiotic use and reduce the burden of antibiotic resistance in Chinese children

**DOI:** 10.3389/fpubh.2024.1530018

**Published:** 2024-12-19

**Authors:** Zhilong Xue, Yanzhi Zheng, Lina Wei, Tong Tian, Jiaqi Wu, Aning Jin, Liping Sun

**Affiliations:** ^1^College of Chinese Medicine, Changchun University of Chinese Medicine, Changchun, China; ^2^College of Clinical Medicine, Changchun University of Chinese Medicine, Changchun, China; ^3^Center of Children's Clinic, The Affiliated Hospital to Changchun University of Chinese Medicine, Changchun, China; ^4^Chinese Medicine Physician Qualification Certification Center, National Administration of Traditional Chinese Medicine, Beijing, China

**Keywords:** traditional Chinese medicine, antibiotic, antibiotic resistance, pediatrics, children

## Abstract

Antibiotic resistance is one of the greatest threats to human health, especially children's health. Traditional Chinese medicine encompasses several documented treatments for pediatric infectious diseases. The antibacterial efficacy and potential of traditional Chinese medicine to reverse bacterial resistance are gaining increasing research attention. This study explores the strategies that have been used to implement traditional Chinese medicine to optimize antibiotic use and reduce the burden of antibiotic resistance in children, as well as the challenges encountered. The findings emphasize the necessity for the Chinese government and the health community to take coordinated action, leveraging the unique strengths of traditional Chinese medicine to address the global health challenge posed by antibiotic resistance.

## 1 Introduction

Antibiotic resistance is one of the greatest threats to human health. A recent study investigating the global burden of antimicrobial resistance showed that an estimated 1.14 million deaths occurred as a result of antibiotic resistance in 2021, and this number is expected to reach 1.91 million by 2050 (1). Antibiotic overuse and misuse are the primary drivers of antibiotic resistance. Therefore, identifying methods to optimize antibiotic use and reduce the burden of antibiotic resistance is crucial.

A recent survey showed that the prevalence of antibiotic use in Chinese children is high. Specifically, the overall prevalence of antibiotic use among outpatients was 63.8%, among inpatients was 81.3%, and at home was 37.8% (2). The 2023 National Antimicrobial Resistance Surveillance Report showed that the detection rates of erythromycin-resistant *Streptococcus pneumoniae* and methicillin-resistant coagulase-negative staphylococci in children were 88.1% and 75%, respectively (3). Notably, the prevalence of erythromycin-resistant Streptococcus pneumoniae in children was higher than in adults and older adults (3). Given the special physiological characteristics of children, antibiotic abuse and misuse not only increase the burden of antibiotic resistance, but they also lead to adverse events and drug toxicity (4). Therefore, optimizing antibiotic use and reducing antibiotic resistance in children has become an urgent problem that needs to be addressed (5, 6). In line with the objectives of the World Health Organization's Global Action Plan on Antimicrobial Resistance, the Chinese government attaches great importance to the issue of antimicrobial drug resistance.

## 2 Current strategies of the Chinese government

Traditional Chinese medicine (TCM) is one of the popularly applied health resources across the globe (7). The antimicrobial effects of Chinese herbal medicines and their ability to facilitate the reversal of bacterial drug resistance have become international research hotspots in recent years (8). Chinese researchers have collaborated with the Karolinska Institute in Sweden and the University of Southampton in the United Kingdom to conduct research on the application of TCMs to address the issue of global antibiotic resistance (9, 10).

China has an original advantage in TCM, which is a scientific and technological resource (11). The Chinese government strongly promotes research that contributes to improving the understanding of the role of TCMs in reducing antibiotic use and antimicrobial resistance in children. With the support of the National Key Research and Development Program of China, TCM researchers have conducted demonstration studies on the use of TCMs to reduce antibiotic use for bacterial infectious diseases in children (12). The aim of this demonstration study was to clarify the efficacy and mechanism of action of TCMs and to use this understanding to formulate guidelines for the clinical application of TCMs and produce guiding principles for the co-administration of antibiotics with TCMs. Additionally, with funding from various TCM research projects, TCM researchers have conducted a series of clinical studies, showing that TCM can effectively reduce the reliance on antibiotics and decrease antibiotic resistance in children (13–24) (Table 1).With respect to clinical guideline development, the National Administration of Traditional Chinese Medicine has actively promoted the formulation of 11 diagnosis and treatment plans for dominant pediatric diseases using TCM (25), as well as four guidelines for the clinical application of Chinese patent medicines in treating these diseases (26–29) (Table 2). Furthermore, the China Association of Chinese Medicine has published the “Clinical Practice Guideline on Traditional Chinese Medicine Alone or Combined with Antibiotics for Patients with Acute Upper Respiratory Infection in Children” (30). Additionally, the World Federation of Chinese Medicine Societies has initiated the development of “Clinical Guidelines on Traditional Chinese Medicine Alone or Combined with Antibiotics for Children with Bacterial Acute Tonsillitis, Acute Bacterial Infectious Diarrhea, Acute Bacterial Infectious of the Lower Urinary Tract, and Bacterial Pneumonia” (31).

**Table 1:** Clinical efficacy of TCM: exemplified by the treatment of respiratory diseases in children.

**Classification**	**TCM treatment method**	**Clinical efficacy**
Using TCM alone	Chinese herbal compound (13)	**Acute bronchitis-caused cough in children**: Reduces cough severity, relieves both daytime and nighttime coughs, alleviates thirst and sputum symptoms, and accelerates cough resolution.
	Acupuncture (14)	**Acute purulent tonsillitis in children**: By regulating cellular immunity, it can decrease the level of inflammation in patients and expedite the resolution of symptoms and signs.
	Cupping therapy (15)	**Cough**:Significant improvement in clinical symptom scores.
	Scrape therapy (16)	**Upper respiratory tract infections in children**:Significant fever reduction.
	Infantile massage (17)	**Recurrent respiratory infections in children**: Reducing the number of episodes, relieving clinical symptoms, enhancing humoral and cellular immune functions, and reducing recurrence rates.
	Sachet (18)	**Childhood pneumonia susceptible groups**:Improvement of symptoms during the recovery period of pneumonia, promotion of physical rehabilitation, and reduction of recurrent respiratory infections in people susceptible to pneumonia.
TCM combined with antibiotics	Chinese herbal compound (19)	**Community-acquired pneumonia in children**: Improves clinical symptoms, shortens the course of the disease, reduces the need for antibiotics, and is safe for clinical application.
	Acupuncture (20)	**Community-acquired pneumonia in children**: Alleviating children's clinical symptoms and reducing the use of antimicrobials and the number of hospitalization days.
	Cupping therapy (21)	**Bacterial pneumonia in children**: Significantly reduces the number of days required for complete remission of fever in children.
	Scrape therapy (22)	**Community-acquired pneumonia in children**: Immediate and sustained antipyretic efficacy.
	Infantile massage (23)	**Mycoplasma pneumonia in children**: Improvement of clinical symptoms, restoration of lung function, and significant reduction in the adverse effects of drugs.
Integrated TCM and Western medicine	TCM comprehensive therapy (24)^*^	**Community-acquired pneumonia in children**: This treatment is effective in improving the signs of pulmonary wet rales in children, and it offers better safety and economy compared to the Western medicine treatment plan.

^*^TCM comprehensive therapy include Chinese herbal medicine compound oral, external application of Chinese herbal medicine (Chinese herbal medicine acupoint application, Chinese herbal medicine umbilical therapy, and Chinese herbal enema), and TCM techniques (Infantile massage and cupping therapy).

**Table 2:** The treatment of dominant diseases with TCM/Chinese patent medicines.

**Classification**	**Disease**
TCM treatment of dominant diseases (25)	Bronchiolitis
	Mycoplasmal pneumonia
	Acute tonsillitis
	Mesenteric lymphadenitis
	Viral myocarditis
	Purpura nephritis
	Acute glomeru-lonephritis
	Primary nephrotic syndrome
	Neurogenic frequent micturition
	Hand-foot-mouth disease
	Infectious mononucleosis
Chinese patent medicine treatment of dominant diseases	Neonatal jaundice (26)
	Acute upper respiratory infection (27)
	Recurrent respiratory tract infections (28)
	Diarrhea (29)

In terms of policy support, the Chinese government formulated the Implementation Plan for Promoting Traditional Chinese Medicine in the Field of Maternal and Child Health (2021–2025) (32), which encourages the strengthening and optimization of TCM in the field of pediatrics. It encourages the screening of diseases for which TCMs may have significant advantages and good clinical efficacy. Moreover, the plan promotes the use of the “Guidelines for the Use of Traditional Chinese Medicine Medical Techniques and Chinese Patent Medicines in Pediatrics”(33) and supports the in-depth fusion of disease prevention using TCM with children's healthcare services. The Action Plan to Improve Child Health (2021–2025) has been issued (34), which focuses on strengthening TCM services for children and promoting TCM healthcare for children, both within households and in the community as a whole. Continuing to develop and refine the National Action Plan to Contain Antimicrobial Resistance (2022–2025) (35), emphasizes the urgency of strengthening research to develop antibiotics that are suitable for children. Moreover, it specifically mentions the importance of developing Chinese patent medicines as alternative antimicrobials.The Opinions on Promoting the High-quality Development of Children's Medical and Health Services has also been issued (36), giving thorough insights into the distinctive advantages of TCM in safeguarding children's health.

## 3 Challenges

Fully harnessing the benefits of TCM in safeguarding children's health still faces challenges. The latest China Statistical Yearbook of Chinese Medicine indicates that there are only 0.518 practicing (assistant) TCM physicians per 1,000 people in China (37), but this number is too small to effectively meet the healthcare needs of children. A national survey evaluating the healthcare service capabilities of grassroots physicians revealed that the competency rate for providing TCM services among these physicians is only 52.7% (38), indicating that the capacity for TCM services at the grassroots level is relatively low. Moreover, the health promotion and popularization of TCM are insufficient, as recent survey data indicate that the level of TCM health literacy of Chinese citizens in 2023 was only 24.62%, with television remaining the primary channel for disseminating TCM health knowledge to the general public (39). In addition, among the seven published clinical practice guidelines on TCM alone or combined with antibiotics to treat common infectious diseases, only one is specific to children (30). Therefore, the existing clinical guidelines are not sufficient to meet clinical needs.

## 4 Future directions

To fully leverage the unique advantages of TCM in reducing antibiotic use in children and lessening the burden of antibiotic resistance, the Chinese government and health community must take coordinated action. First, the size of the TCM workforce should be continuously expanded, and more TCM professionals, especially TCM pediatricians, should be trained to meet the health needs of children with respect to the provision of TCM. Second, the training of grassroots physicians in the knowledge and skills required for the provision of TCM should be strengthened. Moreover, appropriate technology for facilitating the delivery of TCM to children should be promoted. Additionally, the capacity of grassroots medical and health institutions to provide children's TCM services should be strengthened, and children's TCM health services that are equivalent in quality between urban and rural areas should be developed. Third, channels for the dissemination of TCM health knowledge need to be innovated, and new media should be fully leveraged to emphasize the advantages of TCM in the context of children's health among citizens and the community, which would in turn increase the awareness and acceptance of TCM. Moreover, TCM health culture should be comprehensively incorporated into the curricula of primary and secondary schools to cultivate healthy living concepts and lifestyle habits among students. Finally, the development of more clinical practice guidelines on the use of TCM alone or in combination with antibiotics for the treatment of common infectious diseases in children should be prioritized to standardize the clinical use of TCM as a therapeutic approach.

## 5 Conclusion

TCM plays an irreplaceable role in optimizing antibiotic use and reducing the burden of antibiotic resistance in children. The joint efforts of the Chinese government and the health community will help promote the use of TCM, bringing it to the front line of primary healthcare for children. This will be conducive to lowering healthcare costs, reducing the reliance of physicians on antibiotics when treating pediatric diseases, and preventing the emergence of an even more widespread problem of antibiotic resistance. TCM is not only highly important for enhancing the health of Chinese children, but it also contributes positively to the vigorous improvement of children's health worldwide.

## Data Availability

The original contributions presented in the study are included in the article/supplementary material, further inquiries can be directed to the corresponding authors.
